# Prophylaxis of Macular Edema with Intravitreal Ranibizumab in Patients with Diabetic Retinopathy after Cataract Surgery: A Pilot Study

**DOI:** 10.1155/2011/159436

**Published:** 2011-06-16

**Authors:** Patricia Udaondo, Maria Garcia-Pous, Salvador Garcia-Delpech, David Salom, Manuel Diaz-Llopis

**Affiliations:** ^1^Department of Retina and Uveitis, Nuevo Hospital, Universitario y Politecnico La Fe, Valencia, Spain; ^2^Universidad CEU Cardenal Herrera, Edificio Seminario, s/n, Moncada, 46113 Valencia, Spain; ^3^Faculty of Medicine, University of Valencia, 46010 Valencia, Spain

## Abstract

The purpose of this study was to evaluate the effectiveness of intravitreal ranibizumab (Lucentis, Genentech, South San Francisco, Calif, USA) combined with cataract surgery for the prevention of clinically significant macular edema (CSME) in patients with diabetic retinopathy (DR). This prospective interventional case series included fifty-four eyes of 54 patients with a previous diagnosis of nonproliferative diabetic retinopathy (NPDR) without macular edema preoperatively. Subjects were assigned in a 1 : 1 ratio to receive an intraoperative intravitreal ranibizumab injection (*n* = 27) or not (control group, *n* = 27) associated with standardised phacoemulsification surgery. The main outcome measure was the incidence of CSME one and three months after surgery. One month after surgery the incidence of CSME in the control group was 25.92% and 3.70% in the treatment group and at three months was 22.22% and 3.70%, respectively. Short-term results suggest that intravitreal ranibizumab immediately after phacoemulsification prevents CS ME in patients with NPDR.

## 1. Introduction

Diabetic macular edema (DME) is a major cause of central visual loss in diabetic patients [1]. It has been well established by many authors that DME after cataract surgery can difficult visual improvement [2, 3]. 

Shah and Chen [4] suggested that there is no clear evidence that phacoemulsification surgery causes progression DME, particularly in patients with low-risk or absent diabetic retinopathy or in those with controlled retinal disease. However, Kim et al. [5] suggested that 22% of diabetic patients develop increases >30% in central retinal thickness measured by optical coherence tomography (OCT) after uncomplicated phacoemulsification. 

Many studies proposed the used of intravitreal bevacizumab or intravitreal steroids after phacoemulsification in patients with DME to prevent the increase in retinal thickening and to improve the DME and final visual acuity [6–8].

In this study, we injected Ranibizumab, effective in the treatment of wet macular degeneration and macular edema [9–12], immediately after phacoemulsification in diabetic patients with some degree of diabetic retinopathy without macular involvement and studied the macular thickness and morphology by OCT 1 and 3 months after surgery.

## 2. Material and Methods

This prospective, randomized, and controlled intervention study included a total of 54 eyes in 54 patients with cataract and some degree of diabetic retinopathy. All patients were enrolled from December 2009 to September 2010 and were followed up for 3 months. The study protocol was approved by the Ethics Committees of the centre. In addition, this study has been performed in accordance with the ethical standards laid down in the 1964 Declaration of Helsinki for research involving human subjects.

Patients were given an information sheet with the study details, and signed specific informed consent forms for inclusion in the study, cataract surgery and intravitreal injection respectively. 

Inclusion criteria were aged over 18 years and able to make decisions, reduced visual acuity secondary to lens sclerosis and some degree of diabetic retinopathy without macular involvement. Exclusion criteria were previous DME treated or not, any kind of complication during the surgery, other ocular pathology with macular involvement uncontrolled hypertension, recent myocardial infarction, and cerebral vascular accident.

Patients undergoing standard cataract surgery (phacoemulsification with the Infiniti Vision System and monofocal intraocular lens (IOL) implantation) were consecutively assigned in a 1 : 1 ratio to receive an intravitreal injection of ranibizumab (0.5 mL of solution at 10 mg/mL) at the end of surgery or not (control group). All surgeries and intravitreal injections were performed by the same surgeon at the Department of Ophthalmology, University Hospital La Fe in Valencia. Postoperative treatment was identical for all the patients and consisted of the topical administration of tobramycin-dexamethasone eye drops four times a day for one month.

### 2.1. Clinical Examination

A complete ophthalmologic examination was performed before, 1 and 3 months after cataract surgery by the same observer in all cases. The examination included slit lamp examination of the anterior and posterior segment, visual acuity with and without correction, goldmann tonometry, and measurement of the central foveal thickness by OCT retinal examination. Briefly, all eyes underwent macular cube (512 × 128) scans with the Cirrus HD-OCT system (Carl Zeiss Meditec, Dublin, Calif, USA). The central subfield thickness was calculated from the mean of three consecutive measurements. The data obtained in the control and Ranibizumab groups before, 1 and 3 months after surgery, were expressed as the mean  ±  S.D., and compared using the *z*-test.

The main outcome measured was the presence of clinically significant macular edema (CSME) defined as macular edema involving or threatening the centre of the macula as defined by the Early Treatment Diabetic Retinopathy Study [13].

## 3. Results

A total of 54 eyes of 54 consecutive patients with no DME measured by OCT were enrolled in the study in a ratio 1 : 1, 27 eyes in each group. All patients completed the study at 1 and 3 months after surgery. Thirty-five women and 19 men were included, being equivalent the proportion of patients of either sex included in both groups. The mean patient age was also similar in the control and Ranibizumab groups (68.9  ±  4.7 years versus 72.8  ±  5.2 years, resp.). All the patients had a mild to moderate nonproliferative diabetic retinopathy without any differences between control and Ranibizumab groups. The clinical characteristics of patients with diabetes are summarized in Table 1.

### 3.1. Central Macular Thickness (Table 2)

The mean central thickness at baseline was 202 *μ*m in the control group and 206 *μ*m in the Ranibizumab group, being no statically significant.

Seven eyes (25.92%) in the control group presented a CSME measured by OCT compared to 1 in the Ranibizumab group (3.70%) the first month after the surgery. At month 3, 1 patient experienced an improvement in the macular thickness in the control group, so the final percentage of patients that developed a CSME after cataract surgery was 22. 22% in the control group (6 patients) compares to 3. 7% (1 patient) in the Ranibizumab group after 3 months of follow up. 

**Figure 1:**
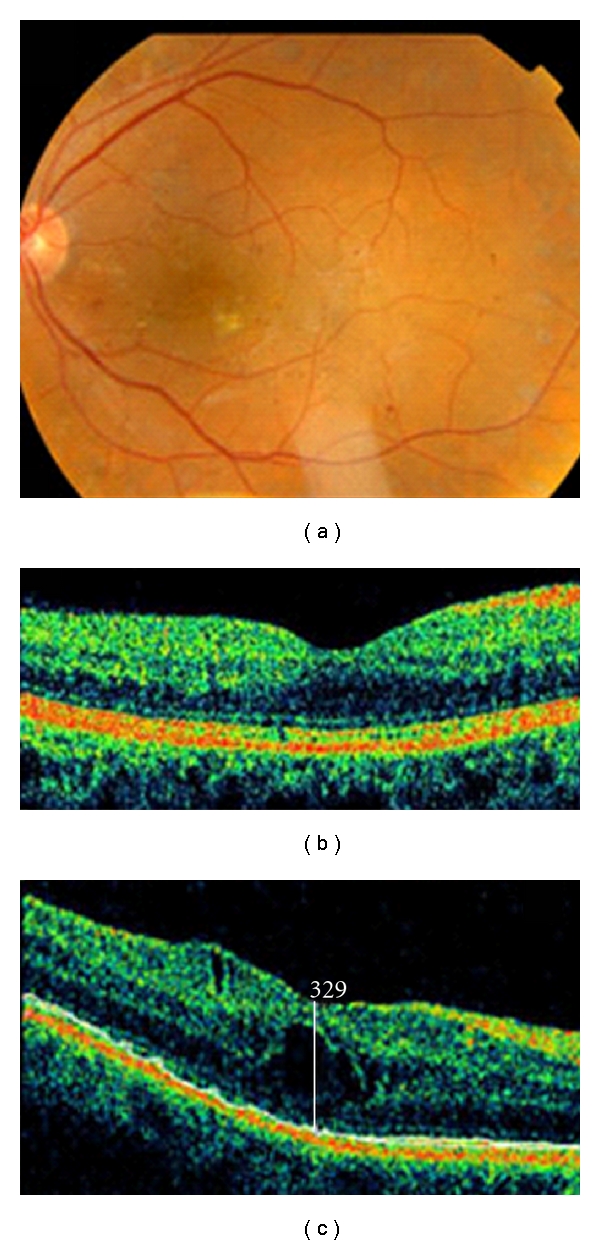
(a) fundus image corresponding to a patient enrolled in the control group that did not receive the prophylaxis after the cataract surgery (b), (c): OCT images of this patient showing the important increase of macular thickness after the cataract surgery without any prophilaxis.

The mean central macular thickness increased from 201 ± 7.2 *μ*m before the surgery to 315,7 ± 36,8 *μ*m at 1 month and 298.4 ± 39.14 *μ*m at 3 months considering the 7 patients that developed macular edema in the control group (Figure 1). It is a mean increase of 105 *μ*m compared to baseline in the patients that developed macular edema. 

The eye in the Ranibizumab group with CSME after phacoemulsification (baseline macular thickness of 198 *μ*m) improved its central thickness from 312 *μ*m 1 month after the surgery to 263 *μ*m at month 3, but it was still clinically significant (Final total increase of 65 *μ*m at the end of the follow up). 

No local or systemic adverse effects were observed in the Ranibizumab group.

## 4. Discussion

This study is, to our knowledge, the first one to report the effectiveness of Ranibizumab in the prevention of CSME after phacoemulsification in patients with mild to moderate diabetic retinopathy.

It has been reported that the VEGF, induced by ocular hypoxia, is an important factor in the development of abnormal angiogenesis and macular edema [14]. Ranibizumab, a recombinant, humanized, monoclonal antibody Fab that neutralizes all active forms of VEGF-A, has recently demonstrated its effectiveness for the treatment of diabetic macular edema [15]. Previously, some reports confirmed the usefulness of Bevacizumab, a humanized monoclonal antibody that inhibits all isoforms of VEGF, in the prevention of macular thickening after phacoemulsification [6, 7, 16]. Bevacizumab is a molecule designed to treat the colon cancer, and Ranibizumab has been specially designed to treat ocular pathology, so the use of Bevacizumab in ocular pathology can be controversial. The success with Bevacizumab in previous studies together with this off-label use of the drug in ophthalmology prompted us to investigate whether the prophylactic use of Ranibizumab in diabetic patients could prevent the development of CSME.

In our results, 25.92% of the eyes in the control group develop a macular thickening which results in a CSME at 1 and 3 months postoperatively. These results are slightly higher to that found by Kim et al. [5], who concluded that 22% of diabetic patients developed increases of the center point thickness of >30% 4 weeks after uncomplicated phacoemulsification. The main difference between both studies is that only patients with mild to moderate diabetic retinopathy were included in our study, and Kim et al. also included patients with none diabetic retinopathy. 

The mean central thickness increased in 105 *μ*m in the 7 eyes of the control group. This value is similar to that published by many series in diabetic patients after phacoemulsification [6, 16, 17]. In the treatment group only 1 patient develop a CSME with an increase in the central thickness of 65 *μ*m, being statically significant the differences between both groups.

Limitations of our study include the short duration of followup, which precludes the determination of the long term safety and efficacy of prophylactic Ranibizumab in cataract surgery and the number of patients included in the study. However, CME develops shortly after cataract surgery with a maximum at 4–6 weeks postop [18]. Our study determined the incidence of CSME at 1 and 3 months, and there was no evidence that new cases of macular edema had developed in the following months if we consider that there were no new cases appearing between the first and the third month after the cataract surgery. 

Although further investigation with a longer followup and a larger series of patients may be needed, the intravitreal injection of Ranibizumab immediately after the phacoemulsification and intraocular lens implantation may be a potent tool to prevent clinical significant macular edema after cataract surgery according to our results.

## 5. Conclusions

In summary, the combination of intravitreal Ranibizumab and uncomplicated phacoemulsification avoids the macular thickening measured by OCT in mild to moderate diabetic retinopathy patients without previous macular involvement. Although the small number of patients and the short followup, the results seem to be promising with no increasing risk for the patient.

## Figures and Tables

**Table 1:** Clinical characteristics of patients before the cataract surgery.

	Control group	Ranibizumab group
Total eyes	27	27
Female	17	18
Male	10	9
Age	68.9 ± 4.7	72.8 ± 5.2
Duration of diabetes (years)	13.4 ± 6.4	14.8 ± 6.5

**Table 2:** Central-macular thickness at baseline compare to 1 and 3 months after the cataract surgery in both groups (including only patients that developed clinical significant macular edema (*CSME).

	Control group	Ranibizumab group
Patients CSME* at 1 month (%)	7 (25.92%)	1 (3.7%)
Patients CSME at 3 months (%)	6 (22.22%)	1 (3.7%)
CMT** Baseline (*μ*m)	201.72 ± 7.2	198
CMT at 1 month (*μ*m)	315.7 ± 36.8	312
CMT at 3 months (*μ*m)	298.4 ± 39.14	263

**CMT: central macular thickness in patients with macular edema. Not included measurements of all eyes.
